# The Role of Free Radicals in Hemolytic Toxicity Induced by Atmospheric-Pressure Plasma Jet

**DOI:** 10.1155/2017/1289041

**Published:** 2017-06-14

**Authors:** Ku Youn Baik, Yoon Ho Huh, Yong Hee Kim, Jeongho Kim, Min Su Kim, Hun-Kuk Park, Eun Ha Choi, Byoungchoo Park

**Affiliations:** ^1^Department of Electrical and Biological Physics, Kwangwoon University, Seoul 01897, Republic of Korea; ^2^Department of Biomedical Engineering, College of Medicine, Kyung Hee University, Seoul 02447, Republic of Korea; ^3^College of Veterinary Medicine, Chonbuk National University, 79 Gobong-Ro, Iksan, Jeonbuk 54596, Republic of Korea

## Abstract

Atmospheric-pressure plasma (APP) has received attention due to its generation of various kinds of reactive oxygen/nitrogen species (ROS/RNS). The controllability, as well as the complexity, is one of the strong points of APP in various applications. For biological applications of this novel method, the cytotoxicity should be estimated at various levels. Herein, we suggest red blood cell (RBC) as a good cell model that is simpler than nucleated cells but much more complex than other lipid model systems. Air and N_2_ gases were compared to verify the main ROS/RNS in cytotoxicity, and microscopic and spectroscopic analyses were performed to estimate the damages induced on RBCs. The results shown here will provide basic information on APP-induced cytotoxicity at cellular and molecular levels.

## 1. Introduction

Atmospheric-pressure plasma (APP) has received attention as a powerful tool for biomedical applications, such as wound healing and cancer therapeutics [1–4]. APP can generate various kinds of reactive oxygen/nitrogen species (ROS/RNS) at one time, such as OH∙, HO_2_, H_2_O_2_, O_3_, NO, and NO_x_^−^, which are controllable by the plasma conditions and environmental conditions [5–7]. APP can induce various biological responses according to the target cells. For example, immune cells produced cytokines [8], and melanocytes produced melanin in response to APP stimuli, resulting in different biological activities [9]. As another example, APP can preferentially kill some cancer cells, when normal cells overcame the same stress [4]. These active responses of cells to the same molecular stimulus make it difficult to standardize APP. In order to make APP successful in medical applications, exact understanding of the primary events as well as the given cytotoxicity should be obtained, before applying APP in each disease.

Red blood cells (RBCs) have been used as a model to investigate oxidative damage in biomembranes. Mammalian RBCs do not have nuclei, ribosomes, and mitochondria, so they do not have protein synthesis systems and mitochondria-based oxidative reactions [10]. Therefore, the response of RBCs to oxidative stress is rather passive and mainly restricted to the membrane. Their main responses are lipid peroxidation, changes in cellular morphology, protein cross-linking, and consequently hemolysis [11, 12]. Another importance of RBCs is their ubiquitous presence in tissues and also their circulation throughout the whole body. APP treatment cannot avoid influencing RBCs during in vivo therapy. In addition, despite their lack of mitochondria, ROS are continuously produced in the RBCs due to the high O_2_ tension in arterial blood and their abundant heme iron content. RBCs are known to regulate vascular oxidative stress through Fenton reaction or NO scavenging by heme irons. Hence, RBC is one of the simplest model systems for the study of toxicity in vitro and one of the important variables for the elucidation of the redox mechanism of APP action in vivo.

Herein, we demonstrate the differential responses of RBCs to pure N_2_ and air gas APP in hemolytic toxicity through membrane damages. In previous reports, we showed that the N_2_ and air gas APP caused differences in the plasma compositions, liquid interactions, and bactericidal effects. Air plasma generated greater amounts of NO_2_^−^ and NO_3_^−^, while N_2_ plasma generated more NH_4_^+^, and showed higher cytotoxicity [6]. In this study, the changes in the RBC morphology, rheological properties, hemolysis, lipid NMR spectroscopy, Raman spectroscopy, and absorption spectroscopy to APP have been examined. The results shown here will provide basic information on APP-induced cytotoxicity in cell and molecular levels.

## 2. Materials and Methods

### 2.1. The Atmospheric-Pressure Plasma Device and Its Characteristics

**Figure 1:**
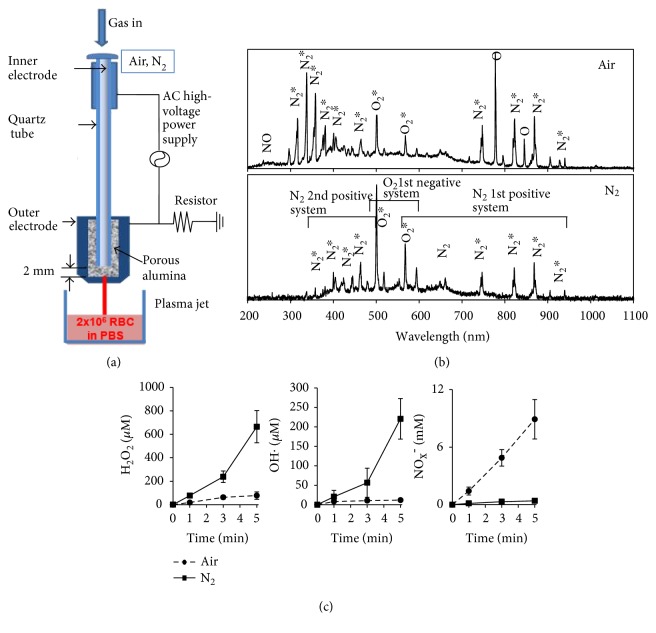
Schematics of nonthermal atmospheric-pressure plasma jet (APPJ) with dielectric porous ceramic and the comparison of the plasma composition between N_2_ and air-feeding gases. (a) A schematic of our experimental system. (b) Optical emission spectroscopy of the plasma plume. (c) Colorimetric analysis of H_2_O_2_, OH, and NO in plasma-treated PBS solution.

Figure 1(a) shows our nonthermal plasma jet system at atmospheric pressure, consisting mainly of a high-voltage power supply, electrodes, and dielectrics. Porous alumina, which has a porosity of 30% and a pore diameter of 100 *μ*m, is used as a dielectric medium between stainless steel electrodes to induce microdischarges and reduces the gas temperature. A more detailed description can be found in previous reports [6, 13]. Voltage of 80 V at 60 Hz was input into a commercial transformer, resulting in an output frequency of approximately 10 kHz and an output voltage of about 1 kV. The output voltage and current waveforms have a sawtooth profile, with an average power of 3 W. The air and N_2_ gas (99.9%) flow rate was 1.0 l/min, and a plasma jet with a plume was ejected into the open air through a 1 mm hole. Spectroscopic measurements were performed with a charge-coupled device (CCD) spectrometer (HR4000, Ocean Optics) and optical fiber (QP400-2-SR) with a diameter of 400 *μ*m. The signal was accumulated for five minutes.

### 2.2. Measurement of H_2_O_2_, OH, and NO in PBS Solution

Immediately after treating phosphate-buffered saline (PBS: LB001-02, Welgene) with atmospheric-pressure plasma jet (APPJ), the concentrations of H_2_O_2_, OH∙, and NO_x_^−^ (NO_2_^−^ and NO_3_^−^); temperature; and pH were measured. The concentration of H_2_O_2_ was determined with Amplex Red reagents (A22188, Molecular Probes), following the manufacturer's protocol. 20 mM terephthalic acid (TA, 185361, Aldrich) solution was directly exposed to APPJ, and the fluorescence intensity was quickly measured. The concentration of NO_x_^−^ was measured with a nitric oxide colorimetric assay kit (K262-200, BioVision), following the manufacturer's protocol. The fluorescence and color changes were measured by a plate reader (Synergy HT, BioTek). The standard curve of OH radical was made with the solutions of different ratios of 20 mM hydroxyl-terephthalic acid (752525, Aldrich) in 20 mM TA solution. Considering TA binding affinity for OH was 35% with O_2_, the measured OH radical concentration should be higher. pH was measured using a pH meter (Eutech Instruments, Singapore). pH of PBS was not changed by 5 minutes of APPJ treatment (data not shown). The temperature of the solutions increased with APPJ exposure. It reached around 35°C when room temperature was 25°C.

### 2.3. Preparation of RBC and Lipid Extraction from Canine Whole Blood

The blood was collected from Beagles and stored in a vacuum bag with anticoagulant CPDA-1 at 4°C before use. The RBCs were purified from the whole blood using Histopaque®-1077 (10771, Sigma). The serum was washed out with 1× PBS (pH 7.0) by centrifugation at 1500 rpm for 10 min. Washing with PBS was repeated for 5 times, and 1 × 10^8^ cells/ml in PBS was prepared as a stock solution. For APPJ treatment, 1 ml of 2% of stock in PBS solution was put in a 24-well plate placed under 3 mm from the outer electrode.

For hemolysis test, the cell suspension was centrifuged at 5000 rpm for 10 min. The absorbance of the supernatant was measured by a plate reader (Synergy HT, BioTek) at 415 nm, because hemoglobin has maximum absorbance there and absorbance between 500 and 700 nm was changed by APPJ treatment. The absorbance of PBS only and RBC in 10% Triton X-100 solution was considered 0% and 100% hemolysis, respectively.

For lipid extraction, 4 volumes of 0.2% ice-cold acetic acid were added and vigorously vortexed. The homogenate was incubated in ice for 5 min, with occasional shaking, then centrifuged at 2500 rpm for 10 min at 4°C, and the supernatant containing intracellular components was removed. New ice-cold 0.2% acetic acid solution was added and centrifuged. This step was repeated more than 5 times to fully remove the intracellular contents. 10 volumes of methanol-chloroform (2 : 1, vol/vol) solution were added to the RBC ghost suspension, and the mixture was vortexed for 5 minutes. 0.5 M KCl in 50% methanol was added, vortexed, and centrifuged. The upper layer was removed, and the step was repeated two more times. The lower layer was dried and resolved in proper solvents for further analysis [14].

### 2.4. Measurement of RBC Deformability

We used Rheoscan-AnD300 (RheoMeditech, Seoul, Korea) to measure the deformability of the RBCs treated with air or N_2_ plasma jet over different exposure times, that is, 1, 3, and 5 min. This ektacytometer uses a laser diffraction technique with microfluidic rheometry and incorporates a disposable microchip [15]. After plasma exposure, the canine RBC suspension was centrifuged at 1700 g for 5 min and washed two times with PBS. Six microliters of the packed RBCs were then mixed with 0.5 ml of polyvinylpyrrolidone (PVP) solution with a viscosity of 30 centipoise at 37°C. The RBC suspension was then injected into a microchip (K-02; RheoMeditech). Test samples flow through a microchannel and experience vacuum-driven shearing. The deformation of the RBC is analyzed by obtaining diffraction images over a range of shear stresses. The captured images of the RBCs were analyzed to calculate the elongation index (EI), defined as (*L* − *W*)/(*L* + *W*), where *W* and *L* are the maximum and minimum axes of the ellipse, respectively.

### 2.5. Raman Spectroscopy, NMR Spectroscopy, and Absorption Spectroscopy

The chemical changes of RBC were analyzed using optical absorption spectroscopy, Raman spectroscopy, and NMR spectroscopy. For the optical spectroscopy, RBC suspension was used directly, and the absorption was read in the plate reader (Synergy HT, BioTek) from 300 to 700 nm at 10 nm intervals. For Raman spectroscopy, the slide glass was cleaned with piranha solution (H_2_SO_4_:H_2_O_2_ = 3 : 1) and coated with gold of 20 nm thickness (Ti 100/Au 200 Å) by thermal evaporation. RBC and lipid extracted from RBC was dropped onto the gold surface and dried in air. The chemical bonding properties were examined by Raman spectroscopy (Alpha300, WITec) with a ×50 object lens and an acquisition time of 10 s. Three different wavelengths including 488 nm, 532 nm, and 633 nm were used with the laser power of 1 mW. For the ^1^H NMR measurement, the liquid extracted from RBC was recovered with CDCl_3_, and the ^1^H NMR spectra were recorded by Bruker AVANCE 600 MHz (14.1 T) spectrometry using a 5 mm TXI cryoprobe. The NMR spectra are assigned with reference to the NMR lipid database. Antioxidants used for optical spectroscopy were purchased from companies as follows: catalase (CAT; C3556, Sigma), superoxide dismutase (SOD; S9636, Sigma), carboxy-2-phenyl-4,4,5,5-tetramethylimidazoline-1-oxyl 3-oxide (cPTIO, 81540, Cayman), and terephthalic acid (TA; 185361, Aldrich). The units of the enzyme activities for CAT and SOD were calculated according to the properties supported by the company. One unit of CAT can decompose 1.0 *μ*M of H_2_O_2_ per minute at pH 7.0 at 25°C. One unit of SOD can inhibit the rate of reduction of cytochrome c by 50% in a coupled system using xanthine and xanthine oxidase.

## 3. Results and Discussion

### 3.1. Reactive Species Generated from APPJ according to Feeding Gases

In our experimental condition, air and N_2_ APPJ generated various types of ROS/RNS in gas states. In optical emission spectroscopy, spectra related to N_2_-excited molecules were dominant in both the air and N_2_ APPJ (Figure 1(b)). The N_2_ second-positive system (C^3^Π_u_-B^3^Π_g_) was typically accompanied by the N_2_ first-positive system (B^3^Π_g_-A^3^Π_u_^+^), which indicated the formation of N_2_ A^3^Σ_u_^+^ long-lived metastable states as reservoirs of energy-promoting plasma chemical reactions. However, atomic O spectra dissociated by higher energy transfers were observed only in air plasma, which was consistent with previous reports showing a higher gas temperature in O_2_ plasma than in N_2_ plasma. The number of electrons was decreased by attachment during the formation of negative O^−^ and O_2_^−^ ions, and the lifetimes of active molecules were also found to be shorter in air plasma, due to collisional quenching by oxygen.

To understand the biological effects of APPJ in solutions, H_2_O_2_, OH·, and NO_x_^−^ were quantitatively analyzed (Figure 1(c)). All the values increased with the increase of APPJ treatment time. The amounts of H_2_O_2_ and OH· were measured much higher in the solution treated with N_2_ APPJ, while the amount of NO_x_^−^ was measured much higher in the solution treated with air APPJ. The higher amount of H_2_O_2_ and OH· may be ascribed to higher electron density in N_2_ APPJ, since the electrons in the air plasma plume easily attach to oxygen species. Electrons hydrolyze water molecules, forming various submolecular species out of water molecules, such as hydrogen atoms and hydroxyl radicals. The higher amount of NO_x_^−^ species may be ascribed to higher atomic O and NO molecules in air APPJ. These characteristics may be connected to different chemical reactions caused by air and N_2_ plasma.

### 3.2. Morphological Changes and Hemolytic Toxicity of RBCs

**Figure 2:**
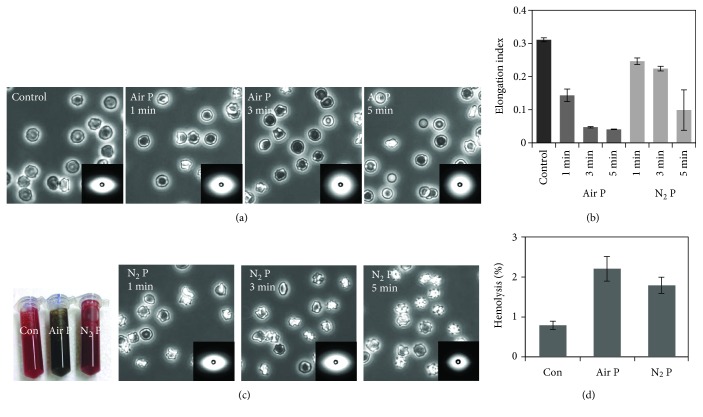
Morphological changes of RBCs by air and N_2_ APPJ treatment, and their hemolytic phenomena. (a) Optical microscopic images with small inserted images showing deformability under shear force. (b) Measurement of deformability. (c) Photograph of RBC solutions by air and N_2_ plasma treatment for 5 min. (d) Measurement of hemolysis of RBC solutions by air and N_2_ plasma treatment for 1 min.

In order to investigate the effects of respective air and N_2_ APPJ treatment on mammalian cell membrane, we exposed canine red blood cells (RBCs) in PBS solutions. The healthy RBC has biconcave morphology, as shown in Figure 2(a) “Control,” which gives elastic and biorheological properties. The shape and deformability are essential features of their biological functions. Figure 2(a) “Air P” and “N_2_ P” show the morphological changes of RBCs in response to the APPJ treatment for 1, 3, and 5 min with air and N_2_ gases, respectively. With air APPJ treatment, RBCs were shrunken after 1 min, and finally became round after 5 min. In contrast, with N_2_ APPJ treatment, RBCs became spiculated with plasma exposure time. These different morphological changes should be attributed to different chemical components in the solutions coming from air and N_2_ plasma.

It has been known that a variety of agents can modify the shape of RBCs [16]. One set of agents, including anionic amphipaths, high salt, high pH, ATP depletion, and cholesterol enrichment, induces spiculated shapes, called echinocytes. Another set of agents, including cationic amphipaths, low salt, low pH, and cholesterol depletion, induces concave shapes, called stomatocytes. Further loading with both types of agents induces budding off the membrane, resulting in spherical bodies. Our air APPJ treatment induced RBCs to form stomatocyte-like structures, while N_2_ APPJ treatment induced echinocyte-like structures. Considering previous findings, air APPJ might reduce the area of outer membrane or increase the area of inner membrane, while N_2_ APPJ might do the opposite [16].

As RBC morphology determines the rheological properties, their deformability was measured after APPJ treatment.  Figure 2(b) demonstrates an elongation index (EI) representing RBC deformability determined from the diffraction image of RBCs at a shear stress of 3 Pa. The shape of RBCs when exposed to air plasma jet tends to become gradually spherical, which indicates a reduction in RBC deformability. The EI values of the RBCs exposed to the air plasma for 1, 3, and 5 min were 54%, 85%, and 87%, which provide lower values of deformability than the control sample (EI = 0.311), respectively. The EI values of N_2_ plasma-exposed RBCs at 1, 3, and 5 min were 0.010, 0.007, and 0.061, respectively, which were 21%, 28%, and 68% lower values of deformability than the control sample (0.311). In general, the EI values of plasma-exposed RBCs at different exposure times were lower than those of the control sample that had an elliptic shape. We observed that the RBC membrane hardness exposed to N_2_ plasma was less than that exposed to air plasma at each exposure time. A similar reduction of EI value was also reported in a previous study with a dielectric barrier discharge [17].

In addition, we observed differential color changes of RBCs after air and N_2_ APPJ treatment (Figure 2(c)), which implied the oxidation of intracellular hemoglobins (Hbs). This intracellular oxidation implies the penetration of ROS/RNS through the membrane. In order to examine the effects of APPJ on membrane integrity, we examined the hemolysis by measuring the absorption of Hbs leaked out from RBCs (Figure 2(d)). While the values increased in both gas APPJ treatments, air APPJ induced more leakage in the RBC membranes. In previous studies, RBCs from hemolytic anemia patients were measured to have increased membrane stiffness, as well as reduced deformability [18, 19]. Therefore, we tried to confirm the increase of elastic moduli of RBCs by using AFM nanoindentation methods. However, the elastic moduli were estimated larger in air APPJ-treated RBCs, but smaller in N_2_ APPJ-treated RBCs, compared to normal RBCs (Supporting Information, Figure S1 available online at https://doi.org/10.1155/2017/1289041). These values were measured by point indentation by sharp AFM cantilever, so they could be different from the EI value, which represents whole-cell structural deformation. The echinocyte-like structures acquired after N_2_ APPJ treatment may be related to reduced volume or inner pressure, which might be related to the reduced elasticity. Stomatocyte-like structures acquired after air APPJ treatment may have opposite characteristics.

In summary, both air and N_2_ APPJ reduced deformability and increased hemolysis in a time-dependent manner. Air APPJ showed more reduction in those measurements for the same exposure duration, which implied the membrane integrity was damaged more severely with N_2_ and O_2_ simultaneous discharges. Their differential changes in morphology and color suggest differential chemical reactions in these two conditions.

### 3.3. Characterization of Chemical Changes of RBC Lipid—NMR Spectroscopy

**Figure 3:**
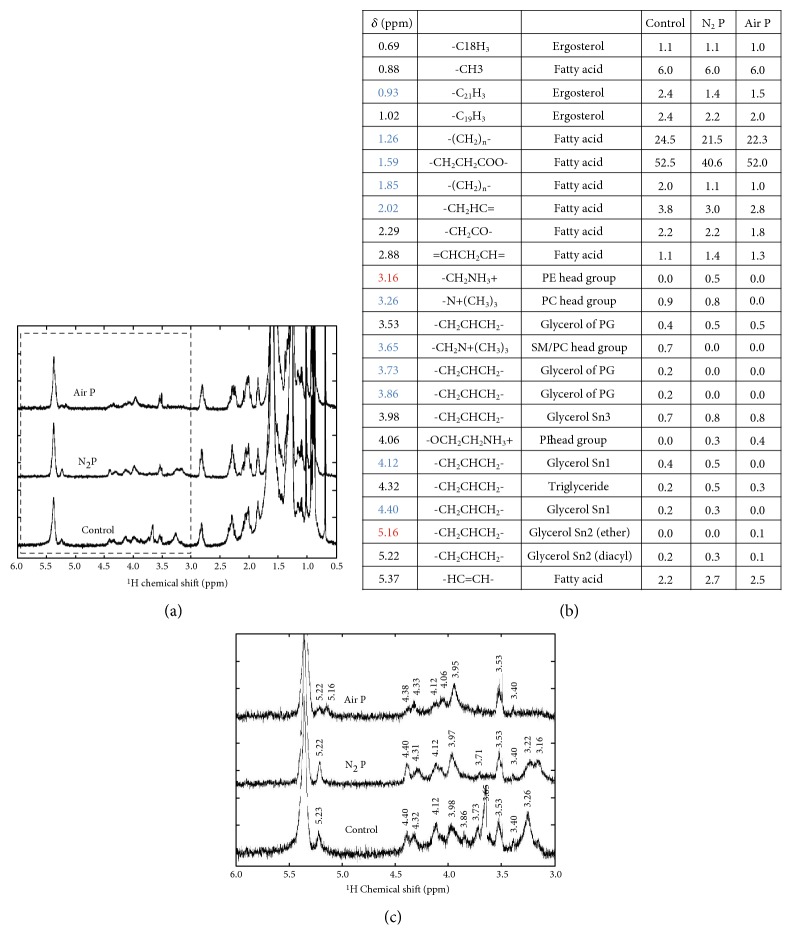
NMR spectroscopy of RBCs after air and N_2_ APPJ treatment. (a-b) NMR spectroscopy of RBC lipid extracts in the range between 0.5 and 6.0 ppm and 3.0 and 6.0 chemical shifts, respectively, and (c) assignments of chemical shifts and the proton number for each peak analyzed by peak area.

Though damages in cytoskeletal proteins, such as spectrin, actin, and band 3, could also lead to structural deformation [20], we first focused on the chemical changes in membrane lipids, because of their vulnerability to oxidative stress. The ^1^H NMR spectra of the total lipid extracted from the RBCs show detailed molecular changes after 5 min of APPJ treatment [21, 22].  Figure 3(a) shows NMR spectra in the range of 0.5 and 6.0 ppm, since there were no clear peaks over 6 ppm chemical shift. The peaks of control, N_2_ plasma-treated, and air plasma-treated RBCs were overlapped well below 3.0 ppm, where the peaks were generally attributed to fatty acids and ergosterols. The peak areas were integrated, proton numbers being normalized by the peak area at 0.88 ppm representing [−C**H**_3_] as 6, and are shown in the table of Figure 3(b). The proton numbers at 1.26 ppm representing [−(C**H**_2_)_n_-] were reduced after both N_2_ and air APPJ treatment from 25 to 22. This means the reduction of fatty acid chain length by plasma treatment. There were more significant reductions at 1.59 ppm representing [−CO-CH_2_-C**H**_2_-], where the number of protons was measured as 53, 41, and 52 for control, N_2_ plasma-treated, and air plasma-treated RBCs, respectively. This implies the chemical changes of glycerol or fragmentation of phospholipids between fatty acid and glycerol. It is notable that N_2_ APPJ caused more severe effects on these changes.

Figure 3(c) shows the spectra between 3.0 and 6.0 ppm, where the positions of the peaks are largely changed. Some peaks have almost disappeared (marked with blue numbers in Figure 3(b)), and some have become relatively dominant after plasma treatment (marked with red numbers in Figure 3(b)). Peaks in this range are generally attributed to protons at phospholipid head groups and glycerol backbones. The chemical shift value of each peak is marked on the graph. In both N_2_ and air APPJ treatments, peaks at 3.26 and 3.65 ppm are significantly reduced, which represent ^1^H at the choline [−N(C**H**_3_)_3_] of phosphatidylcholine or sphingomyelin. In contrast, peaks at 3.16 ppm are significantly enhanced after only N_2_ APPJ treatments, which represents [−C**H**_2_NH_3_^+^] of phosphatidylethanolamine. Chemical reactions might possibly occur between the phospholipids and ammonium ions from N_2_ APPJ.

Peaks between 3.73 and 5.22 ppm are generally related to the glycerol backbone, which connects the head and tail groups of the phospholipid. Most peaks remain after the plasma treatment; however, their relative ratio has changed. Peaks at 3.73 and 3.86 ppm are reduced by both plasma conditions, representing phosphatidylglycerol. However, peaks at 4.12 and 4.40 ppm are significantly reduced by only air plasma condition, representing glycerol Sn1 methylene protons. Sn1 carbon is connected to one part of acyl chains as Sn2 carbon, but it is more exposed to outer attacks, such as ROS from APPJ. In addition, a peak at 5.16 ppm newly appears in the case of air plasma treatment, which represents glycerol Sn2 protons connected to ether lipids. It is also notable that the peak at 5.37 ppm representing unsaturated fatty acid is almost conserved, and no significant peaks at 9.7 and 11 ppm representing aldehyde or carboxyl groups separately are observed.

In summary, our results imply that the outer head groups of the phospholipids or glycerols are vulnerable to the plasma treatment, as compared to the fatty acid tail groups inside the membrane, especially to air APPJ. The high power of removing the head group of phospholipids of air APPJ may result in reduction of the outer leaflet and following stomatocyte-like distortion. Likewise, the addition of ammonium ions on the head group may be the reason for echinocyte-like distortion of RBCs by N_2_ APPJ.

### 3.4. Characterization of Chemical Changes of RBC—Raman Spectroscopy

**Figure 4:**
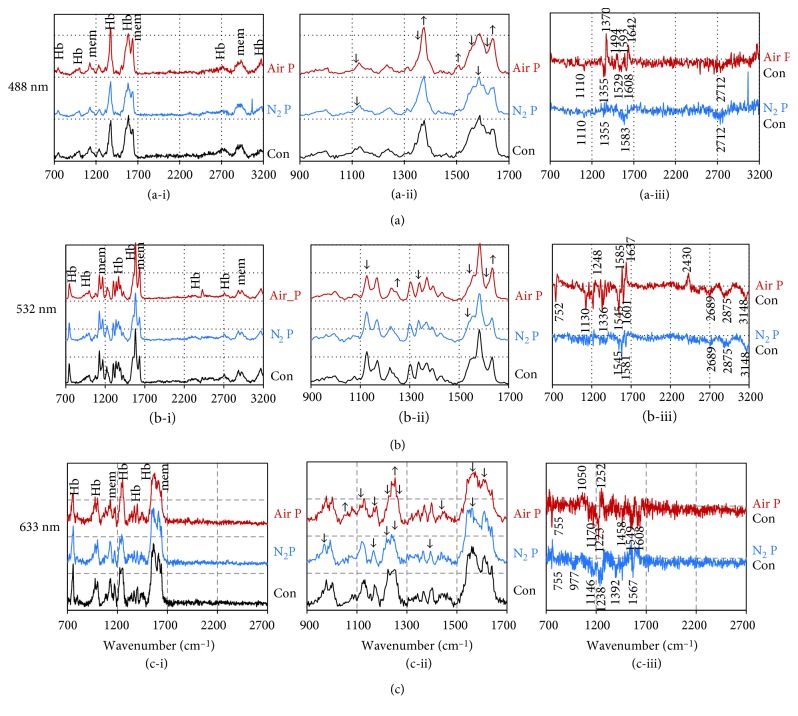
Raman spectroscopy of RBCs after air and N_2_ APPJ treatment using excitation lasers with wavelengths of (a) 488 nm, (b) 532 nm, and (c) 633 nm. The Raman spectra of control RBCs, air APPJ-treated RBCs, and N_2_ APPJ-treated RBCs were displayed in the ranges of (i) 700–3200 cm^−1^ and (ii) 900–1700 cm^−1^ wave numbers, and (iii) the differences between APPJ-treated samples and control were separately displayed.

In order to obtain chemical changes in whole RBCs, we tried Raman spectroscopy with 488, 532, and 633 nm lasers. Different excitation wavelengths bring differential Raman spectra of RBCs, due to the specific absorption bands of RBCs [23]. In previous reports, the main Raman spectral bands of RBCs were mostly assigned to hemoglobin vibration energy, and some were to cell membrane lipids and proteins [23, 24].  Figure 4 shows the Raman spectra of RBCs after 5 minutes of APPJ treatment. The main bands were from hemoglobin (marked with “Hb” in the figure) in the region of 1340–1380 cm^−1^ corresponding to various pyrrole ring vibration modes and 1500–1640 cm^−1^ corresponding to the combination of spin state marker vibration modes. Peaks at 752 cm^−1^ and 798 cm^−1^ correspond to ring breathing, while peaks at 934 cm^−1^ and 674 cm^−1^ correspond to ring symmetric stretch and deformations of Hb. The protein bands (marked with “mem” in the figure) include mainly the amide I band at 1648 cm^−1^ and several low peaks of CH_2_/CH_3_ deformation modes primarily from amino acid side chains at 1448 cm^−1^, CH_2_ twist mode at 1324 cm^−1^, skeletal vibrations at 973 cm^−1^, and C-C stretch of phenylalanine ring at 998 cm^−1^. The lipid bands (marked with “mem” in the figure) include the acyl chain stretch modes at 2850–3000 and 1060–1180 cm^−1^ and CH_2_/CH_3_ deformation modes at 1448 cm^−1^.

Figure 4(i) shows that both air and N_2_ APPJ treatment induced slight intensity changes, rather than frequency modulations.  Figure 4(ii) shows enlarged graphs in the region of 900–1700 cm^−1^, and Figure 4(iii) shows graphs that reveal deviations of the spectra between plasma-treated samples and control, respectively. The peaks whose differences are noticeable are marked with up or down arrows in Figure 4(ii), and center wavenumber values are marked in Figure 4(iii).

In the case of 488 nm laser (Figure 4(a)), the peak at 1110 cm^−1^ became lower (corresponding to acyl chain stretch↓), the peak at 1370 cm^−1^ became sharper and higher (corresponding to pyrrol ring vibration: 1355 cm^−1^ representing Fe^2+^↓ and 1375 cm^−1^ representing Fe^3+^↑), and the wide band of multiple peaks at 1550–1650 cm^−1^ (1530 cm^−1^ for C = C Trp↓, 1545 cm^−1^ for Fe^2+^↓, 1608 cm^−1^ for Fe^2+^↓, and 1642 cm^−1^ for Fe^3+^↑) were modified by air APPJ treatment [24]. The peak at 2712 cm^−1^ was an overtone of 1355 cm^−1^, whose intensities reduced together. N_2_ APPJ treatment reduced peaks at 1583 cm^−1^, which also represented the reduction of Fe^2+^-Hb.

With 532 nm laser (Figure 4(b)), the spectral changes were similar to those with 488 nm laser. Acyl chain length may be shortened (1130 cm^−1^↓), and iron of Hb may be oxidized to Fe^3+^ (1545 and 1601 cm^−1^ Fe^2+^↓ and 1637 cm^−1^ Fe^3+^↑) by air APPJ treatment. In addition, the sharp reduction at 752 cm^−1^ was related to the reduction of porphyrin breathing mode of Hb, which represents the viability of RBCs [25]. The enhancement of peak at 1248 cm^−1^ was related to the protein denaturation/heme aggregation, and that of the peak at 2430 cm^−1^ was related to the increase of hydrogen bonds of O-H…O type [26, 27]. The reduction in 2875–2915 cm^−1^ represented the reduced amount of CH_2_ or CH_3_ groups of acyl chains. Raman spectra of RBCs treated by N_2_ APPJ shows less difference to control, which means less impact on the lipid membrane and the Hb spin state by N_2_ APPJ.

With 633 nm laser (Figure 4(c)), the spectra patterns of air and N_2_ APPJ-treated RBCs showed the most difference from each other. As observed with 488 and 514 nm lasers, air APPJ treatment shortened the acyl chain length (1150–1170 cm^−1^↓), and reduced O_2_-bound Hb (1223, 1549, and 1608 cm^−1^↓). Air APPJ treatment also enhanced the peaks at 1252 cm^−1^, which is rather strongly observed in met-Hb. N_2_ APPJ treatment also reduced the acyl chain length (1150–1170 cm^−1^↓). In addition, N_2_ APPJ treatment enhanced the peaks at 1549 and 1608 cm^−1^ and reduced the peaks at 977, 1238, 1392, and 1567 cm^−1^, which related to the reduction of O_2_-bound Hb [28].

Overall, Raman spectroscopy data show that both gas APPJs can affect the lipid membrane and even modify the spin states of intracellular Hb. It was difficult to discern peaks associated with proteins separately. APPJ reduced the length of acyl chains of lipid membrane, and reduced the O_2_-bound Hbs to O_2_-detached form, or further to met-Hb (Fe^3+^ Hb without oxygen). Additionally, we analyzed them using PCA (Figure S2). This qualitative multivariate analysis helped us to discriminate one sample from the others. We performed PCA for RBC samples exposed to air APPJ, N_2_ APPJ, and H_2_O_2_. Four groups (control, air APPJ, N_2_ APPJ, and H_2_O_2_) seemed to be distinctly classified, but the boundary of control and N_2_ plasma-exposed RBC samples was found to be ambiguous. The result was the same when we tried the same analysis with lipid membrane ghosts from RBCs. These analyses suggest that air APPJ made distinct effects in comparison to N_2_ APPJ or H_2_O_2_, and the N_2_ APPJ made less change in the molecules of RBCs than air APPJ.

### 3.5. Oxidation of Intracellular Hemoglobin—Absorption Spectroscopy

**Figure 5:**
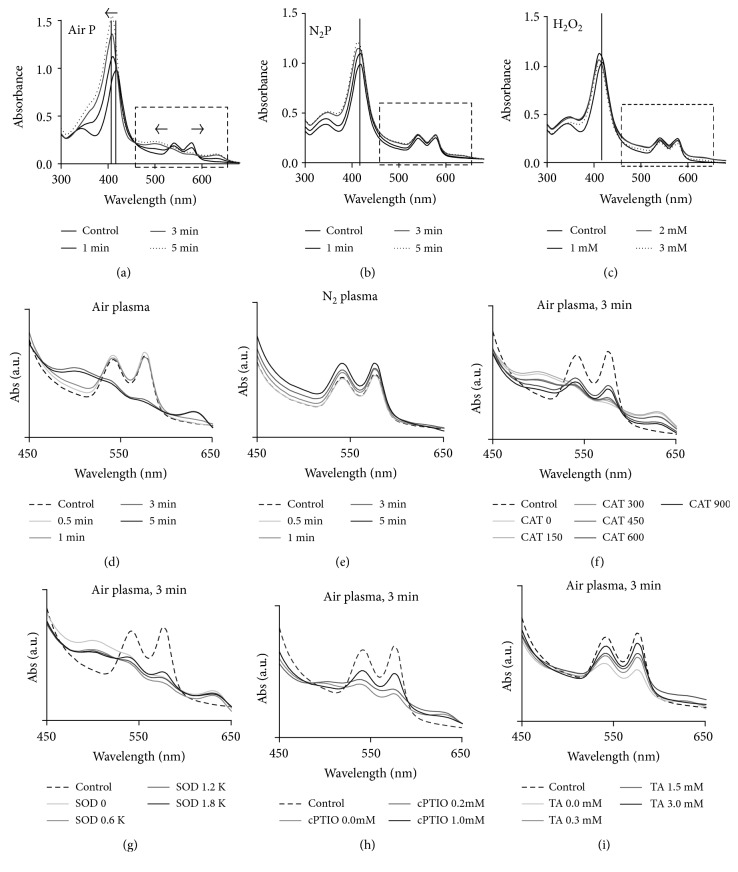
(a–c) Absorption spectroscopy of air and N_2_ APPJ-treated RBCs and H_2_O_2_-treated RBCs, respectively, in the range of 300–650 nm. (d-e) Absorption spectroscopy of air and N_2_ APPJ-treated RBCs, respectively, in the range of 450–650 nm wavelengths. (f–i) Absorption spectroscopy of air APPJ-treated RBCs for 3 min with several antioxidant chemicals.

Hbs in RBCs have specific absorption bands of light, which bring red color to RBCs.  Figure 2(b) shows that the air APPJ-treated RBCs are brownish color, but N_2_ APPJ-treated RBCs are still red. The changes in absorption band denote the oxidation of Hbs in RBCs. Figures 5(a), 5(b), and 5(c) show the optical absorption spectra of RBCs between 300 and 650 nm according to the APPJ treatment time. Oxygen-bound Hb has three main absorption bands at about 415 (soret or B-bands), 540 (Q_v_ or *α* band), and 575 nm (Q_0_ or *β* band) [22]. Air APPJ changes these three peaks severely (Figure 5(a)). The absorption bands are shifted from 415 to 408 nm, from 540 to 499 nm, and from 575 to 630 nm with air APPJ treatment time. These peak shifts are assigned to the Fe^3+^ Hbs without oxygen (met-Hb), which are the oxidized form of Hbs by reactive species from air APPJ [29]. In contrast, the changes by 5 min of N_2_ APPJ or by 3 mM H_2_O_2_ are comparatively negligible (Figures 5(b) and 5(c)). The enlarged absorption spectra in the range of 450 to 650 nm confirm these observations (Figures 5(d) and 5(e)). Air APPJ has the potential to oxidize Hbs inside RBCs to met-Hbs with longer than 3 min exposure, while N_2_ APPJ does not.

In order to examine which reactive species were responsible for the met-Hb formation by air APPJ, we added several antioxidants to the RBC homogenates when they were exposed to air APPJ for 3 min. Figures 5(f), 5(g), 5(h), and 5(i) show the absorption spectra of air APPJ-treated RBCs with catalase (CAT), superoxide dismutase (SOD), carboxy-PTIO (cPTIO), and terephthalic acid (TA), respectively. CAT is a well-known scavenger for H_2_O_2_, cPTIO for NO·, SOD for O_2_∗-, and TA for OH· radicals. The spectra were displayed according to the increase of concentrations of each scavenger in the graphs. All scavengers alleviated the oxidation of RBCs; however, cPTIO and TA seemed more effective. The spectra remained at some level, even with high amount of CAT or SOD, which meant their reducing effects on Hb oxidation were limited to some levels. In contrast, the spectra recovered similar to control when cPTIO or TA was added, which implied NO∙ and OH∙ played major roles in Hb oxidations by air APPJ.

The chemical analysis of APPJ-treated solutions showed that N_2_ APPJ produced more amounts of OH∙ and H_2_O_2_, while air APPJ produced more amount of NO∙ in PBS solutions (Figure 1(c)). This implies that NO∙ plays the most important roles in air APPJ induced intracellular Hb oxidation. Though NO∙ is a relatively stable radical, it still has the power to modify biological molecules. NO∙ reacts rapidly with O_2_-bound Hb, resulting in met-Hbs and nitrate; reacts with Hb, resulting in nitrosyl-Hbs; and reacts with met-Hbs, resulting in Hb and nitric oxide cation [30, 31]. Thus, the lifetime of NO∙ in aqueous solution is about several min, about several sec in tissues, and about millisec in blood vessels [32]. In the case of OH∙ radicals, it would be hard for them to directly interact with RBCs, due to their short lifetime of about nanoseconds. The effects of TA in our experiment may be attributed to scavenging the OH∙ radicals reverted through Fenton reactions from their stabilized molecule H_2_O_2_ [10].

Due to the high affinity of Hbs with NO∙, RBCs have regulatory roles in the modulation of intravascular NO∙. However, intravascular hemolysis releases Hbs into the plasma compartment, resulting in rapid rates of NO∙ consumption. Consequently, vascular relaxation and vasodilation are inhibited. It was reported that administration of blood Hb substitute solutions in clinical trials led to pulmonary and systemic hypertension, increased systemic vascular resistance, decreased organ perfusion and gastrointestinal paresis, and increased rates of death in trauma patients [33]. APP treatment initiates multiple mechanisms through chemical oxidation/nitrosation of biomolecules in anticancer action, DNA transfection, and so forth. However, the RBC hemolysis can induce toxicity in tissue or whole-body damage through Hbs release into tissue or blood flow. Therefore, the exact estimation of cytotoxicity by APPJ should be systemically investigated [34–36].

## 4. Conclusions

In conclusion, both air and N_2_ APPJ have been shown to be able to induce morphological and hemolytic changes of RBCs in a time-dependent manner. Though N_2_ APPJ generates higher amounts of OH∙ and H_2_O_2_ oxidants, air APPJ elicits changes in RBCs at much shorter time through severe damage of the lipid membrane. In addition, air APPJ can alter intracellular O_2_-bound Hbs to met-Hbs, which loses O_2_ carrying potential. NO∙ generated in air APPJ may play critical roles in APPJ-induced cellular toxicity, including hemolysis, and iron-containing molecular oxidations. These results provide a useful biological model to estimate APPJ toxicity and provide insight into APPJ cytotoxicity in biological applications.

## Supplementary Material

Figure S1. Elasticity of APPJ-treated RBCs measured by atomic force microscopy. Figure S2. Raman spectroscopy of RBC and RBC lipid membrane after Air and N2 APPJ treatment and their PCA analysis.
