# Supplementary material for: The Role of Free Radicals in Hemolytic Toxicity Induced by Atmospheric-Pressure Plasma Jet
Source: Oxid Med Cell Longev. 2017 Jun 14;2017:1289041. doi: 10.1155/2017/1289041 (PMC5488234; doi:10.1155/2017/1289041)
Supplement: Supplementary file 1 — Figure S1. Elasticity of APPJ-treated RBCs measured by atomic force microscopy. Figure S2. Raman spectroscopy of RBC and RBC lipid membrane after Air and N2 APPJ treatment and their PCA analysis. [file 1289041.f1.docx]

Supporting information

**Figure S1. Elasticity of APPJ-treated RBCs measured by atomic force microscopy.**


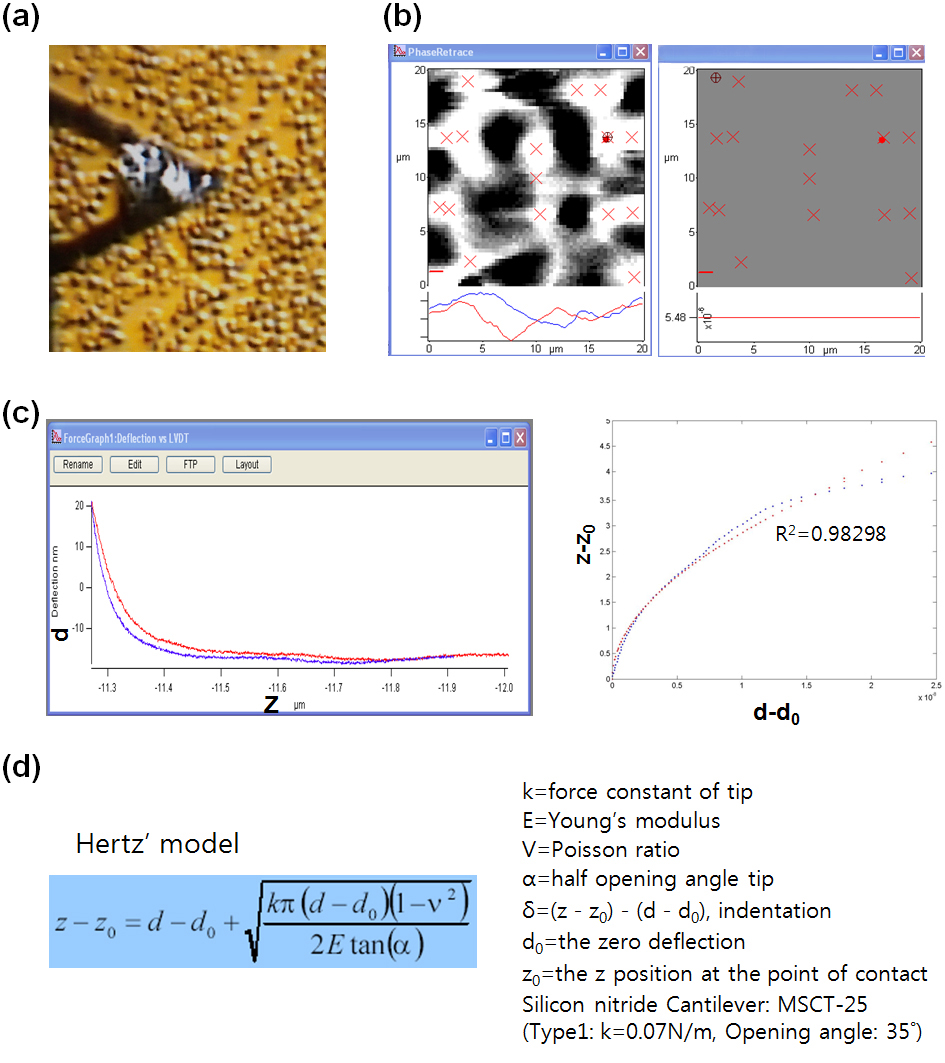


(a) RBC homogenate in PBS solution was dropped onto poly-L-lysine coated glass, and incubated for 30 minutes. After washing with PBS, the attached RBCs were loaded under cantilever.

(b) Because it was difficult to load cantilever exactly on top of the RBCs using videoscope, the morphology was roughly scanned with low loading force at high speed. Positions with higher profile were pointed to, and memorized in the software. RBCs were not detached throughout this process.

(c) The data of force-distance curves were automatically harvested at those positions, and the value at the initial contact point of each curve was subtracted, as shown in the figure.

(d) The z position vs. deflection of cantilever was fitted with Hertz’ model, and the Young’s modulus was acquired when the R^2^ value was over 0.95.


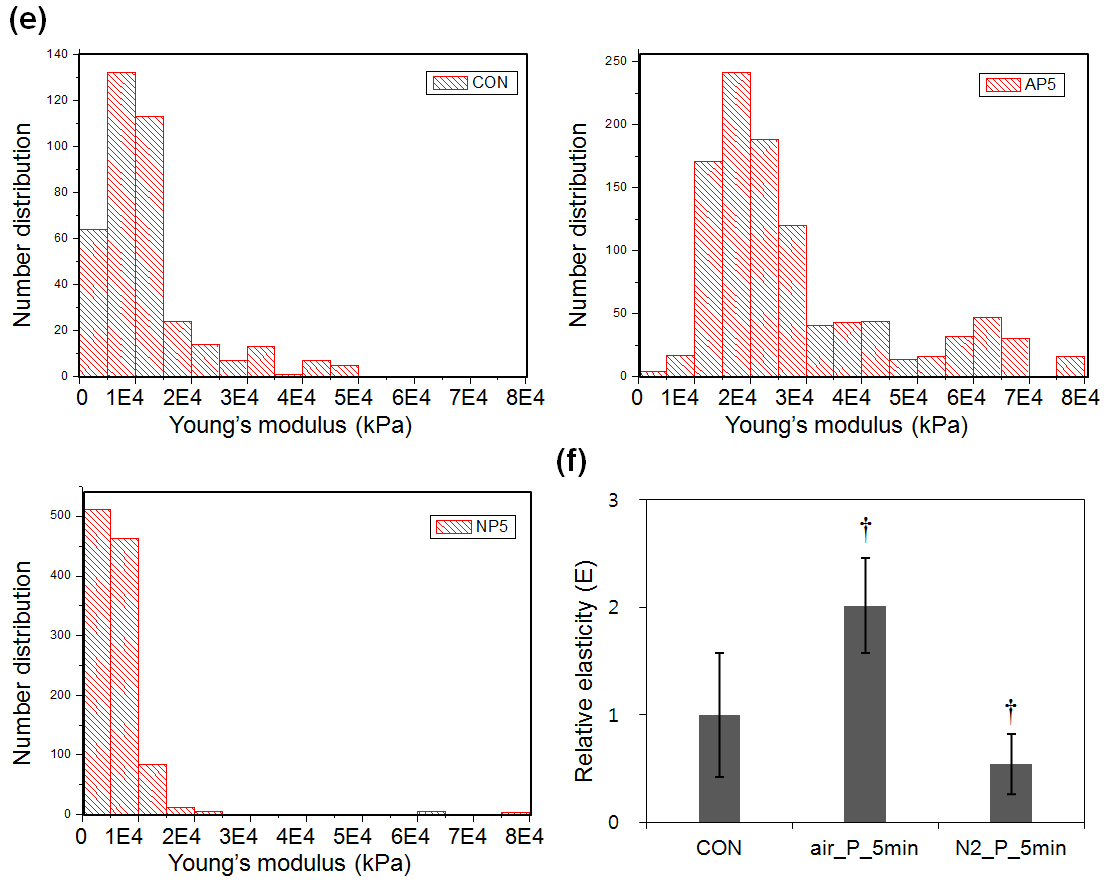


(e) The number distributions of the Young’s modulus of control, air APPJ-treated, and N_2_ APPJ-treated RBCs were plotted separately.

(f) The mean values of Young’s modulus of each sample were plotted with standard deviation. The elastic modulus of air APPJ-treated RBCs increased, and that of N_2_ APPJ-treated RBCs decreased (Student’s T test: p<0.01).

**Figure S2. Raman spectroscopy of RBC and RBC lipid membrane after Air and N_2_ APPJ treatment and their PCA analysis.**


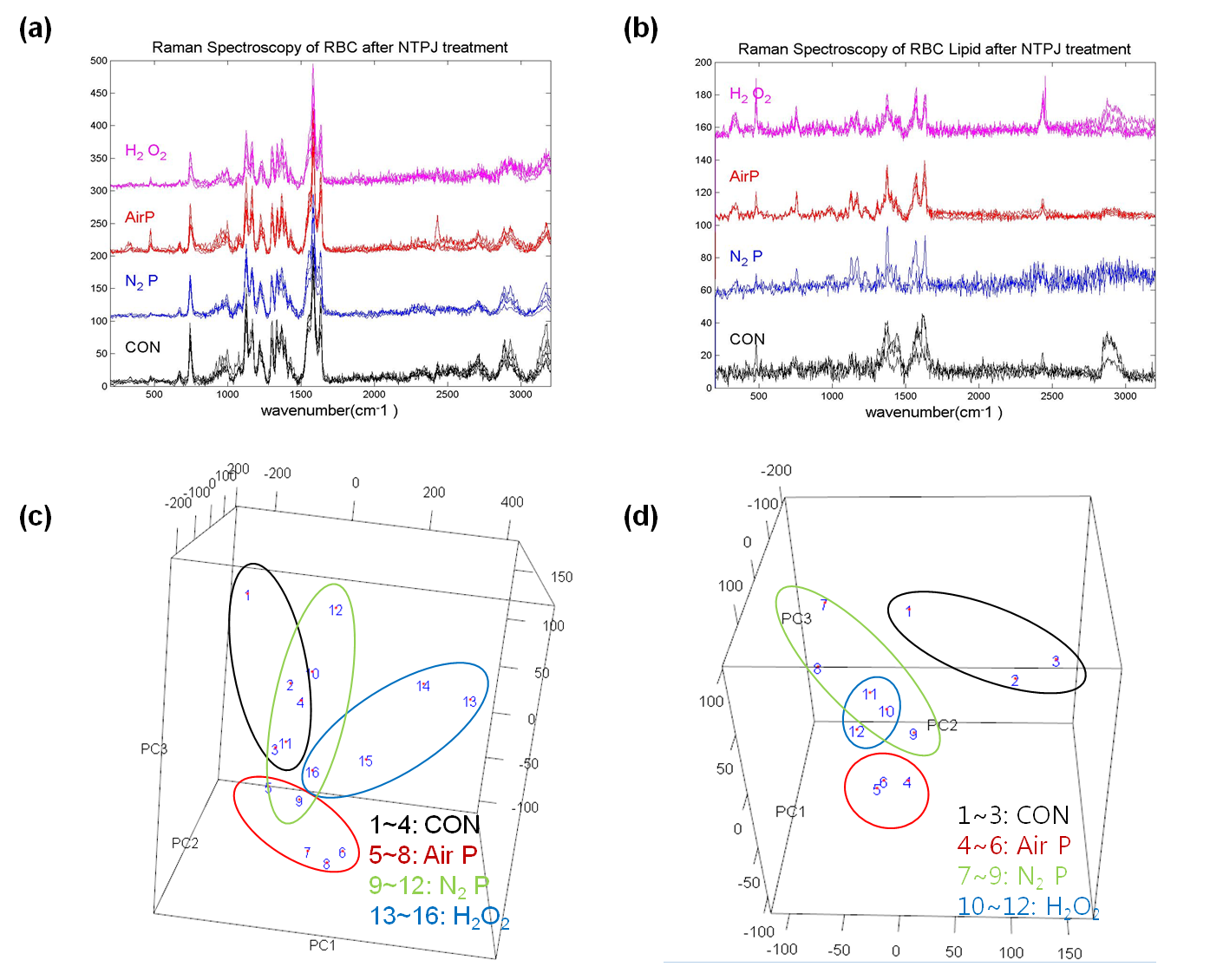


(a–b) Raman spectra of whole RBC and lipid extracts in the range between 300 and 3,200 cm^-1^ wave number were acquired with 633 nm laser. Normal RBCs, RBCs treated with air APPJ for 5 minutes, RBCs treated with N_2_ APPJ for 5 minutes, and RBCs treated with 1 mM H_2_O_2_ for 1 hour were compared [1].

(c–d) The principle component analyses of (a) and (b). The graph was mapped with Matlab, and the differences between samples were analyzed using principal component analysis (PCA) for a two-group classification problem. PCA of the Raman data was performed using the built-in R function *prcomp.* R is a free software environment for statistical computation and graphics [2].

[1] Donald F.H. Wallach, Surendra P. Verma, Raman and resonance-Raman scattering by erythrocyte ghosts, Biochimica et Biophysica Acta (BBA) - Biomembranes 382 (1975) 542-551

[2] The R Project for Statistical Computing. [https://www.r-project.org](https://www.r-project.org/) (Accessed November 4, 2016).
